# Higher Hospital Frailty Risk Score Is an Independent Predictor of In-Hospital Mortality in Hospitalized Older Adults with Obstructive Sleep Apnea

**DOI:** 10.3390/geriatrics7060127

**Published:** 2022-11-14

**Authors:** Temitope Ajibawo, Oluwatimilehin Okunowo

**Affiliations:** 1 Department of Medicine, Banner University Medical Center, Phoenix, AZ 85006, USA; 2 Data Science and Biostatistics Unit, Children’s Hospital of Philadelphia, Philadelphia, PA 19104, USA

**Keywords:** hospital frailty risk score, obstructive sleep apnea, mortality

## Abstract

Background: Frailty predisposes individuals to stressors, increasing morbidity and mortality risk. Therefore, this study examined the impact of frailty defined by the Hospital Frailty Risk Score (HFRS) and other characteristics in older hospitalized patients with Obstructive Sleep Apnea (OSA). Methods: We conducted a retrospective study using the National Inpatient Sample 2016 in patients ≥65 years old with OSA. Logistic regression was used to evaluate the impact of frailty on inpatient mortality. A Kaplan-Meier curve with a log-rank test was used to estimate survival time between frailty groups. Results: 182,174 discharge records of elderly OSA were included in the study. 54% of the cohort were determined to be a medium/high frailty risk, according to HFRS. In multivariable analysis, frailty was associated with a fourfold (medium frailty, adjusted odd ratio (aOR): 4.12, 95% Confidence Interval (CI): 3.76–4.53, *p*-value < 0.001) and sixfold (high frailty, OR: 6.38, 95% CI: 5.60–7.27, *p*-value < 0.001) increased odds of mortality. Hospital survival time was significantly different between the three frailty groups (Log-rank test, *p* < 0.0001). Comorbidity burden defined by Charlson comorbidity Index (CCI) was associated with increased mortality (*p* < 0.001). Conclusion: More than half of the whole cohort was determined to be at medium and high frailty risk. Frailty was a significant predictor of in-hospital deaths in hospitalized OSA patients. Frailty assessment may be applicable for risk stratification of older hospitalized OSA patients.

## 1. Introduction

OSA is a sleep disorder characterized by infrequent partial or complete airway collapse ensuing in episodes of frequent awakenings, loud snoring, choking, and disrupted sleep [1]. These episodes frequently terminate with brief arousals from sleep resulting in the restoration of airway patency [2]. The apnea-hypopnea index (AHI) is used to estimate the incidents of apnea (interruption of airflow for ≥10 s) and hypopnea (decrease in the airflow by greater than 30% for at least 10 s with accompanying reduction in oxygen saturation) [3]. The prevalence of OSA in the United States (US) is about 10–15% in women and greater in men—15–30% [3]. If OSA is defined by AHI as greater than or equal to five episodes per hour, the prevalence of OSA is roughly 5% in females and 15% in males [4]. Obese, older males are at a greater risk of developing OSA, and the prevalence of OSA is higher in African Americans, irrespective of body weight [5,6,7].

The chance of developing OSA increases with age, and the prevalence is two- to threefold higher in older adults older than 65 years compared to younger adults (30–64 years) [8]. Comorbid conditions such as obesity, hypertension, renal failure, stroke, and hypothyroidism are common in older adults and may partly explain the increased prevalence in this population [2].

Subjects with OSA have a high prevalence of hypertension, and people with hypertension, on the other hand, have a high prevalence of OSA [9,10,11]. Continuous Positive Airway Pressure (CPAP) is the therapy of choice in OSA [12].

Aging has been linked to physiological and anatomic changes that make this age group prone to sleep-disordered breathing, such as OSA [2]. On imaging, a few studies have demonstrated changes, such as bony structure changes and fatty deposits in the pharynx walls causing narrower airways that may partially explain the increased prevalence of OSA in older adults [13,14]. Furthermore, the observed higher airway resistance and reduced pharynx dilator muscle activity during sleep compared to younger adults demonstrated in some prior studies have been implicated in the pathogenesis of OSA in older adults [15,16,17]. In addition, older subjects tend to spend a more significant amount of sleep time in stage 1 and stage 2, characterized by higher respiratory instability, which aids periodic breathing and predisposes them to upper airway collapse [18]. Lastly, post-menopausal females have lower estrogen levels leading to further decreased pharyngeal muscle activity and greater airway resistance [11]. The consequences of OSA in older adults include an increased risk of cardiovascular morbidity, stroke, higher limitation in activities of daily living, depression, and worsening cognitive status [19,20,21,22].

Frailty is characterized by diminished physiologic reserve and an increased risk of adverse health events [23]. Frailty affects approximately 17% and 10% of older adults in middle-income and high-income economies, respectively [24]. However, physical frailty varies widely between 7% to 40% in older adults [25,26]. Our study used the Hospital Frailty risk score, a validated, prudent method of frailty assessment, to help in risk stratification and distribution of medical resources [27]. HFRS was derived from data from more than one million older patients in the United Kingdom [27]. HFRS has been used for assessing frailty in other disease conditions such as heart failure, ruptured intracranial aneurysms, and hepatocellular carcinoma [28,29,30]. Although the Fried frailty phenotype is the most used frailty screening measure, we could not use the Fried frailty phenotype because it requires performing physical tests. Our data were obtained from a patient-de-identified database.

The impact of frailty on in-hospital mortality in older adults with OSA is mainly unknown. Therefore, we aim to study the influence of HFRS on in-hospital mortality in hospitalized OSA patients.

## 2. Materials and Methods

### 2.1. Data Source

We utilized the discharge data from the National Inpatient Sample (NIS) for 2016, sponsored by the Agency for Healthcare Research and Quality (AHRQ) [31]. NIS is the largest all-payer inpatient database in the United States, and it contains approximately eight million discharges in a year or 20% of all nationwide hospital discharges [31].

### 2.2. Study Population

The study population was obtained from hospital discharges from the NIS. First, we utilized the International Classification of Diseases, 10th revision, clinical modification (ICD-10) codes of G47.33 to identify hospital discharges of patients with OSA. Patients ≥ 65 years were identified. Frailty was defined by HFRS, a validated ICD-10 coding algorithm developed by Gilbert et al. [27]. The coding algorithm was obtained from a cluster of frail subjects with 109 ICD-10 codes assigned several points ranging from 0.1 to 7.1, reflecting the strength of association to the frail collection [27]. These points were summed for a final frailty risk score. After the calculation of the HFRS scores, patients were classified as low frailty (<5), medium frailty (5–15), and high frailty (>15) risk [27]. Baseline patient characteristics include age, gender, ethnicity, primary payer, admission, bed size, hospital teaching status, and comorbidities using the Charlson Comorbidity Index (CCI). Discharge data with missing data were excluded.

### 2.3. Statistical Analysis

Descriptive analyses were conducted on patient and hospital characteristics. Counts and percentages were used for describing categorical variables. Differences between the three frailty groups were assessed with Pearson’s Chi-squared test, median and interquartile ranges were reported for continuous variables, and differences between the three groups were determined using the Kruskal-Wallis test. Univariate and multivariate regression analyses were used to evaluate the effect of frailty on mortality. In multivariate analysis, the variables of frailty status, age, gender, race/ethnicity, admission status, length of stay and CCI were included in the model to determine the effect of frailty on inpatient mortality. Kaplan Meier estimates with the log-rank test were used to compare survival rates between the three frailty groups. *p* < 0.05 indicated a significant difference. SAS version 9.4 (SAS Institute Inc, Cary, NC, USA) was used for data management, and all statistical analyses were performed using R Version 4.0.4.

## 3. Results

**Figure 1:**
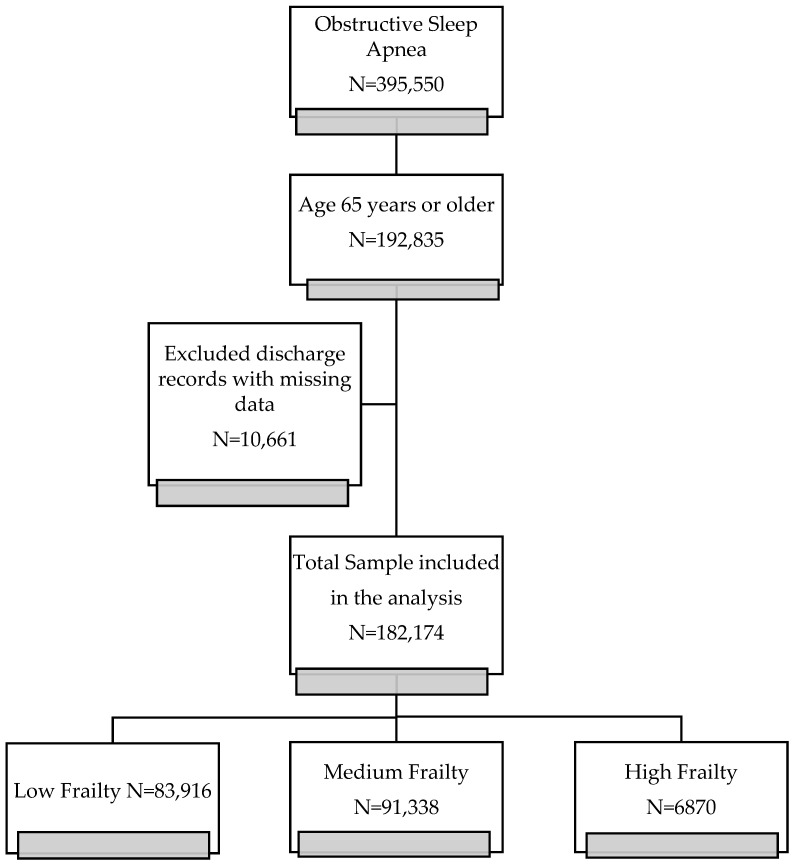
Study Flowchart.

We identified a total sample of 182,174 discharge records of OSA after excluding missing records with missing data. According to HFRS, 83,916 (46%) were classified as low frailty, 91,388 as medium frailty (50%), and 6870 (4%) as high frailty. Figure 1 shows the flow chart demonstrating how the cohort of OSA patients was obtained.

Most patients were white (83%), Medicare beneficiaries (89%), admitted to urban teaching hospitals (66%) and stayed in the hospital for less than seven days (74%). The median Charlson Comorbidity Index score (CCI) among all patients was 3.0 (IQR, 2.0–5.0); the low frailty cohort had the lowest average CCI (median: 2.0, IQR, 1.0–4.0). The most common comorbidities among all patients were chronic pulmonary disease (46%), congestive heart failure (44%), renal disease (36%), and diabetes without complications (32%). The baseline characteristics of the low, medium and high frailty risk groups are shown in Table 1.

In both unadjusted and adjusted analyses, females had lower odds of mortality in older OSA patients during hospitalization (Table 2). However, medium and high frailty were associated with a six and eleven-fold higher risk of inpatient mortality than low frailty (Table 2).

**Figure 2:**
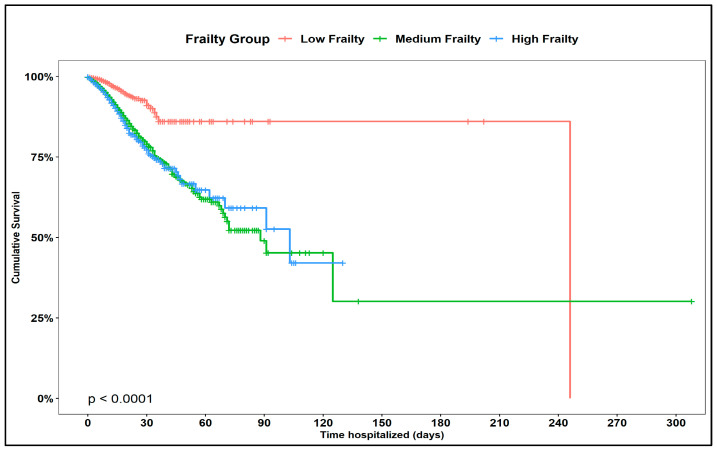
Kaplan-Meier curve estimating the Impact of frailty on inpatient mortality.

Kaplan-Meier estimation with log-rank test showed that survival time was different between the three frailty groups (*p* < 0.0001). The low frailty group had an average survival duration of 246 days compared to 88 days and 103 days for the medium and high frailty groups, respectively (Figure 2).

## 4. Discussion

In this contemporary nationwide study of the US using a validated frailty risk score, the key findings were (1) Frailty was associated with increased mortality risk in older hospitalized OSA patients, and survival time differed significantly between the frailty groups. (2) Frailty is common, with approximately 54% of the whole cohort of older OSA hospitalized patients determined as having a medium or high risk of frailty. (3) Medium and high frailty had higher CCI scores than low frailty risk, and CCI was associated with a higher risk of in-hospital mortality in older OSA patients.

A significant finding of our study was the increased mortality risk among OSA patients with an intermediate or high frailty risk. In multivariable regression analysis, the odds of hospital mortality were fourfold and sixfold in medium and high frailty risk, according to HFRS. Although we did not find any prior study in the literature on the impact of frailty status on mortality risk in older adults with OSA, a study by Ensurd et al. reported a prospective cohort that included 2505 older non-frail men with sleep disturbances [32]. In the study, severe sleep apnea, defined by apnea-hypopnea >30, was associated with mortality risk at follow-up but not increased odds of frailty at follow-up.

Multiple mechanisms may explain the mortality risk observed in intermediate/high-risk frailty. During sleepless nights in OSA, physical stress forces can release inflammatory mediators, such as IL-10 and IL-6, through endothelial activation [33]. In addition, sleep deprivation can modify the biomarkers of stress response like norepinephrine or cortisol. An increased amount of these biomarkers can prompt in vitro production of cytokines such as interleukin-6 (IL-6) and TNF-α [33,34,35]. Pro-inflammatory markers indirectly induce frailty by modifying major metabolic pathways or through increased proteolysis [36].

Furthermore, the pro-inflammatory environment can increase oxidative stress by creating and releasing oxygen radicals [37]. Consequently, oxygen radicals can produce an arrhythmogenic media leading to ventricular arrhythmias and sudden death [37]. In addition, the inflammatory milieu created can cause coagulopathy, making these patients prone to thrombotic events [38,39]. Frail patients often take longer to recuperate from an illness, given their lower physiological reserves [40,41]. Thus, these patients may stay longer in the hospital and are predisposed to adverse outcomes of hospitalization, such as venous thromboembolism and hospital-acquired infections [41]. The resulting hospital complications from a more extended hospital stay may partially explain why prolonged hospital stays greater than or more than seven days are at increased risk of mortality in our study.

In our study, the median CCI score was higher in medium and high-risk frailty than in low-risk frailty (4 vs. 2, *p* < 0.001). In univariate and multivariate analysis, CCI was associated with a higher risk of mortality (odds ratio-1.13, CI 95%: 1.12–1.15, *p* < 0.001). OSA is one of the common causes of secondary hypertension and a typical precursor to hypertension [42]. Hypertension affects approximately 7.5 in 10 adults over age seventy, and frailty subsequently complicates the control of blood pressure adjustments [43]. The incidence of hypertension rises with age and OSA [44,45]. The possible mechanisms by which OSA causes hypertension include blood pressure elevation and variability at night, elevated sympathetic activity, increased endothelial malfunction, increased sympathetic- activity, endothelial malfunction oxidative stress, increased arterial stiffness, and repetitive hypoxia [46,47,48,49,50,51]. Arterial stiffness, a sign of functional and structural modification of the vessel wall, is also highly prevalent in hypertension [52,53]. Although the association between arterial stiffness and OSA is not fully understood, hypertensive patients with OSA and arterial stiffness are prone to many adverse events [54].

Furthermore, hyperglycemia is commonly observed in frail older adults with hypertension and is an independent predictor of adverse events in patients without or without diabetes mellitus [55]. For instance, in a study of frail hypertensive adults ≥65 years old, there was a significantly slower five-minute walk test in the hyperglycemia group compared to the normoglycemia group [55]. Therefore, optimal glycemic control may be essential in frail hypertensive patients to decrease functional decline and avoid further adverse events [56,57,58].

Obesity is another comorbid condition commonly associated with OSA [59]. Although multiple mechanisms link OSA and obesity together, how obesity causes OSA is less clear. The proposed mechanisms of causation include: insulin resistance; role of leptin in sympathetic activation; increased levels of inflammatory biomarkers such as IL-6; hypercoagulability; genetics [60,61,62,63,64,65,66,67]. It is well-established that the most important therapeutic intervention for OSA in obese subjects is weight loss [68,69]. Therefore, obese OSA patients should be encouraged to lose weight.

OSA increases the likelihood of developing cardiovascular disease conditions such as heart failure, coronary artery disease, stroke, and arrhythmias such as atrial fibrillation [69]. For example, in a study of community living residents, the Women’s Health Initiative Study, females with coronary artery disease were more likely to develop initial frailty over six years [70]. In another study, the 3C (Three-City), findings revealed that older adults with frailty were more predisposed to cardiovascular events (3.6% versus. 2.8% per year) [71]. In our study, CCI cardiovascular components, which include a history of congestive heart failure and myocardial infarction, were both associated with increased frailty.

The prevalence of cognitive decline rises with age, and it is not uncommon to diagnose physical impairment in older adults [72]. Comorbid conditions such as hypertension, heart failure, and dementia increase the risk of hospitalization, disability, and death [73]. Therefore, it is vital to target the care of underlying comorbid conditions in older adults to reduce cognitive decline and frailty [74,75]

Frailty is a complicated state typical of older adults, which affects physical decline, and managing frailty is a health challenge of rising importance [55]. Therefore, it is imperative that comprehensive geriatric evaluation is one of the best methods for early diagnosis of physical deterioration and management of comorbid conditions [76,77,78,79].

The proportion of patients with intermediate and high-risk frailty was approximately 54%. While we observed that frailty was common amongst older hospitalized OSA patients and linked with reduced survival, modifications in clinical practice to address frailty once identified needs further elucidation. There is evidence of effective interventions to prevent or reduce frailty, including nutritional supplementation, cognitive training, exercise, geriatric assessment, and management [80]. In the setting of OSA, these frail patients, therefore, require personalized care to improve independence and quality of life.

Although there is no gold standard for assessing frailty, there are two significant ways to evaluate frailty: the deficit model, which comprises adding an individual’s conditions and impairments to produce a frailty index, and the frailty phenotype, consisting of 5 domains [40,81]. The Hospital Frailty Risk Score belongs to the deficit model of frailty assessment, and it is utterly dependent on ICD codes [27]. It has also been validated against the Fried frailty phenotype and Rockwood’s Clinical Frailty Scale [27]. As a result of our findings, frailty may be helpful to prognosticate and risk-stratify older adults with OSA.

Caution should be applied while interpreting our study based on some limitations. Firstly, the NIS is an extensive administrative nationally representative database that uses ICD- 10 codes prone to coding errors and misclassification. Secondly, the analysis of NIS is based on hospital episodes with OSA without a patient identifier; therefore, each patient may be counted more than once. Thirdly, residual confounding is possible in any retrospective study; therefore, we cannot demonstrate causality in the relationship between frailty and mortality. Fourthly, a national database like NIS does contain information such as the severity of OSA and prescription medications. For instance, the severity of OSA can contribute to the disparity in outcomes.

Our study involves only hospitalized patients with OSA and no outpatient visits. Consequently, this study’s findings may not be generalizable to outpatients. Furthermore, unaccounted factors such as physical activity level, depressive symptoms, smoking status, and sarcopenia, which are all associated with OSA and frailty, may have attenuated the risk of frailty on in-hospital mortality. Lastly, this study is subject to non-response bias. In identifying our analytic sample, records with missing data on variables like mortality status, gender, race, primary payer, and admissions were excluded. A comparison of excluded discharge records with included records revealed differences in important baseline characteristics and frailty status. The excluded records had a higher proportion of patients with low frailty (47.9% vs. 46.1%, *p* < 0.0001) and a lower proportion of patients with medium (49.0% vs. 50.2%, *p* < 0.0001) and high frailty (3.2% vs. 3.8%, *p* < 0.0001). Although the differences are minimal, they might have resulted in biased estimates of effect.

Our study has some strengths: NIS used a large database that provides valuable information on the impact of frailty on hospitalized older OSA patients throughout the US as it relates to a more significant burden to health services, higher mortality, and loss of independence. In addition, the NIS is a nationally representative database that can account for several confounding factors. We also used a validated hospital risk score (HFRS), which employs hospital administrative data from electronic medical records and is clinically relevant to older patients [27].

## 5. Conclusions

54% of our study cohort of older hospitalized OSA patients were noted to be an intermediate or high-frailty risk. CCI was significantly associated with an increased risk of hospital mortality. Higher HFRS was positively associated with an increased risk of inpatient mortality. Frailty may be helpful in the risk stratification of older OSA patients. Further studies should explore the influence of rehabilitation and CPAP treatment in reducing frailty risk in older OSA patients.

## Figures and Tables

**Table 1:** Baseline Characteristics of Hospitalized OSA Patients.

Characteristics	Overall N = 182,174 ^1^	Frailty Group			*p*-Value ^2^
		Low Frailty N = 83,916 ^1^	Medium Frailty N = 91 388 ^1^	High Frailty N = 6870 ^1^	
Age (years)					<0.001
65–74	106,230 (58%)	54,478 (65%)	48,821 (53%)	2931 (43%)	
≥75	75,944 (42%)	29,438 (35%)	42,567 (47%)	3939 (57%)	
Gender					<0.001
Male	105,127 (58%)	49,536 (59%)	51,927 (57%)	3664 (53%)	
Female	77,047 (42%)	34,380 (41%)	39,461 (43%)	3206 (47%)	
Race/Ethnicity					<0.001
White	151,226 (83%)	70,516 (84%)	75,210 (82%)	5500 (80%)	
Black	17,682 (10%)	7386 (9%)	9474 (10%)	822 (12%)	
Hispanic	8091 (4%)	3666 (4%)	4093 (4%)	332 (5%)	
Asian or Pacific Islander	1858 (1%)	769 (1%)	997 (1%)	92 (1%)	
Native American	652 (0%)	305 (0%)	323 (0%)	24 (0%)	
Other	2665 (1%)	1274 (2%)	1291 (1%)	100 (1%)	
Payer Status					<0.001
Medicare	162,504 (89%)	73,956 (88%)	82,273 (90%)	6275 (91%)	
Medicaid	1274 (1%)	505 (1%)	719 (1%)	50 (1%)	
Private insurance	14,891 (8%)	7780 (9%)	6690 (7%)	421 (6%)	
Self-pay	516 (0%)	237 (0%)	261 (0%)	18 (0%)	
No charge	26 (0%)	14 (0%)	11 (0%)	1 (0%)	
Other	2963 (2%)	1424 (2%)	1434 (2%)	105 (2%)	
Admission Status					<0.001
Non-elective admission	140,307 (77%)	54,063 (64%)	79,878 (87%)	6366 (93%)	
Elective admission	41,867 (23%)	29,853 (36%)	11,510 (13%)	504 (7%)	
Length of Stay (days)					<0.001
<7	135,043 (74%)	72,463 (86%)	59,471 (65%)	3109 (45%)	
≥7	47,131 (26%)	11,453 (14%)	31,917 (35%)	3761 (55%)	
Bedsize of Hospital					<0.001
Small	34,102 (19%)	16,510 (20%)	16,462 (18%)	1130 (16%)	
Medium	52,048 (29%)	23,965 (29%)	26,105 (29%)	1978 (29%)	
Large	96,024 (53%)	43,441 (52%)	48,821 (53%)	3762 (55%)	
Location/teaching Status of Hospital					0.10
Rural	15,141 (8%)	6908 (8%)	7697 (8%)	536 (8%)	
Urban nonteaching	46,659 (26%)	21,350 (25%)	23,521 (26%)	1788 (26%)	
Urban teaching	120,374 (66%)	55,658 (66%)	60,170 (66%)	4546 (66%)	
Charlson Comorbidity Index (CCI) Score	3.0 (2.0, 5.0)	2.0 (1.0, 4.0)	4.0 (2.0, 5.0)	4.0 (3.0, 6.0)	<0.001

^1^ n (%); Median (IQR); ^2^ Pearson’s Chi-squared test; Kruskal-Wallis test.

**Table 2:** Effect of Frailty on Patient Mortality.

Characteristic ^3^	Univariable				Multivariable		
	N	OR ^1^	95% CI ^2^	*p*-Value	OR ^1^	95% CI ^2^	*p*-Value
Frailty Status	182,174						
Low Frailty		—	—		—	—	
Medium Frailty		6.04	5.53, 6.62	<0.001	4.12	3.76, 4.53	<0.001
High Frailty		11.1	9.84, 12.6	<0.001	6.38	5.60, 7.27	<0.001
Age (years)	182,174						
65–74		—	—		—	—	
≥75		1.68	1.59, 1.79	<0.001	1.37	1.29, 1.46	<0.001
Gender	182,174						
Male		—	—		—	—	
Female		0.84	0.79, 0.89	<0.001	0.84	0.79, 0.89	<0.001
Race/Ethnicity	182,174						
White		—	—		—	—	
Black		0.96	0.87, 1.06	0.5	0.84	0.75, 0.93	<0.001
Hispanic		1.01	0.88, 1.16	0.9	0.93	0.81, 1.07	0.3
Asian or Pacific Islander		1.24	0.95, 1.60	0.10	1.01	0.77, 1.31	>0.9
Native American		1.35	0.85, 2.01	0.2	1.38	0.87, 2.08	0.14
Other		1.27	1.01, 1.57	0.031	1.26	1.00, 1.56	0.039
Admission Status	182,174						
Non-elective admission		—	—		—	—	
Elective admission		0.29	0.26, 0.32	<0.001	0.51	0.46, 0.57	<0.001
Length of Stay (days)	182,174						
<7		—	—		—	—	
≥7		2.13	2.01, 2.26	<0.001	1.42	1.33, 1.51	<0.001
Charlson Comorbidity Index	182,174	1.24	1.22, 1.25	<0.001	1.13	1.12, 1.15	<0.001

^1^ OR = Odds Ratio, ^2^ CI = Confidence Interval, ^3^ Variables included in the multivariate analysis.

## Data Availability

Data is publicly available at https://www.hcup-us.ahrq.gov/nisoverview.jsp (accessed on 10 July 2022).
